# Family Intensive Behavioral Intervention for Children with Autism Spectrum Disorder: A Half-Year Comparison-Controlled Study

**DOI:** 10.31083/AP38796

**Published:** 2025-02-28

**Authors:** Yi Wu, Xueping Chen, Duo Li, Hongwu Wang, Yan Ou, Shaoyuan Su, Guimin Liu, Qingying Zhang, Wenwang Rao

**Affiliations:** ^1^Department of Pediatrics, Second Affiliated Hospital of Shantou University Medical College, 515041 Shantou, Guangdong, China; ^2^Department of Preventive Medicine, Shantou University Medical College, 515041 Shantou, Guangdong, China

**Keywords:** autism spectrum disorder, children, family-intensive behavioral intervention

## Abstract

**Objective:**

Family intervention is a crucial component of treatment for children with autism spectrum disorder (ASD), yet the impact of parent-mediated family-intensive behavioral intervention on the language abilities of children with ASD has been barely studied. The purpose is to investigate the effectiveness of the Verbal Behavior Milestones Assessment and Placement Program (VB-MAPP)-based family-intensive behavioral intervention in enhancing the language abilities of children with ASD. This study provides insights to help ASD children better cope with daily life.

**Methods:**

From September 2020 to September 2022, a total of 85 clinically diagnosed children with ASD and 30 age- and sex-matched children without ASD were recruited. Applied Behavior Analysis (ABA) and VB-MAPP were used for evaluating and determining individualized intervention programs for children with ASD. The intervention lasted 6 months.

**Results:**

There were no significant differences in demographic characteristics between children with ASD and children without ASD (all *p *> 0.05), except for the mother’s age. After the intervention, there was a significant increase in all VB-MAPP scores among children with ASD (all* p *< 0.001), compared with the baseline VB-MAPP total score and 16 domain scores. Tests of noninferiority showed that children with ASD at post-intervention were non-inferior to children without ASD in the Visual Perceptual Skills and Matching-to-Sample (VP/MTS) score (*p* = 0.001), play score (*p *= 0.034), reading score (*p* < 0.001), and writing score (*p *< 0.001).

**Conclusions:**

Family-intensive behavioral intervention significantly improved the skills of children with ASD, as assessed by the VB-MAPP. These findings emphasize the importance of family intervention and provide further support for proposing a family intervention program for children with ASD that is suitable for China’s national conditions.

## Main Points

1. Family-intensive behavioral intervention could significantly increase all VB-MAPP scores among children with ASD.

2. Compared with children without ASD, children with ASD had lower the VB-MAPP total score and 15-dimensional scores.

3. The language, social skills, and multifaceted skills of children with ASD could be strengthened using the VB-MAPP-based family-intensive behavioral intervention.

## 1. Introduction

Autism spectrum disorder (ASD) is a highly heritable neurological and developmental disorder that presents with a variety of symptoms; those affected have difficulty communicating and show repetitive behaviors, highly restricted interests, and/or altered sensory behaviors beginning early in life [1, 2]. The prevalence of ASD is relatively high and continues to rise [3]. As shown in a meta-analysis, the global prevalence of ASD among 30,212,757 participants in 74 studies was 0.6% [4]. Children with ASD may have deficient cognitive abilities [5], high levels of intellectual disability [6], and high proportions of co-occurring psychiatric or neurodevelopmental diagnoses [7]. Moreover, children with ASD can cause a heavy economic burden for families and society [8, 9] and use both acute and specialty care more often than their counterparts without ASD [10]. The Global Burden of Disease Study 2019 (GBD 2019) estimated that approximately 43.07 per 10,000 disability-adjusted life years (DALYs) worldwide were attributed to ASD in 2019 [11].

Given the absence of approved drugs for treating the core symptoms of ASD [12], the primary treatment for children with ASD is to minimize the core deficits through professional training (e.g., behavioral intervention, language training, and social skills development) [13]. Many studies have provided evidence for effective intervention approaches in treating ASD, including applied behavior analysis (ABA), music therapy [14], cognitive behavioral therapy [15], and special education [16]. However, certain intervention strategies (e.g., intrathecal autologous bone marrow stem cell therapy and nutritional and dietary interventions) do not yield the desired results [17, 18].

Applied behavior analysis-based interventions are widely considered to be the most effective for children with ASD [19]. Common types of interventions based on ABA comprise discrete trial training (DTT) and natural environment teaching (NET). The DTT program is a teaching technique, which consists of a series of direct and systematic instructional methods that are applied repeatedly until the child has acquired the necessary skills [20]. The NET program is a therapeutic strategy that places an emphasis on learning within a naturalistic and comfortable setting (e.g., at home, school, or the community) [21].

At present, interventions for children with ASD mainly rely on training in rehabilitation institutions and special schools [22, 23], but there are still not enough professionals, and professional capabilities need to be improved. Moreover, professional ABA-based intensive behavioral intervention is expensive and cannot be afforded by the average household [24]. In addition, the COVID-19 epidemic led to the closure of rehabilitation institutions and special schools and the decline of parents’ willingness to enroll their children with ASD in institutional training programs. Thus, to address these shortcomings, ABA-based intensive family behavioral training guided by professionals is worth exploring.

The Verbal Behavior Milestones Assessment and Placement Program (VB-MAPP) is an assessment and placement program for children with ASD, which integrates the procedures and teaching methods of ABA and Skinner’s Verbal Behavior Analysis [25]. The Verbal behavior milestones assessment and placement program-based intervention has received widespread recognition as a promising tool for developing comprehensive early intervention programs [26]. Numerous studies have demonstrated that VB-MAPP-based intervention could significantly improve the multifaceted abilities of children with ASD [27, 28, 29]. The aim of this study was to explore the effectiveness of VB-MAPP-based family-intensive behavioral intervention in enhancing the language abilities of children with ASD. Our results offer valuable insights toward the effective development of family intervention strategies for children with ASD.

## 2. Methods

### 2.1 Subjects

This was a longitudinal intervention study among Chinese children with ASD. All samples were recruited from the Department of Pediatrics at the Second Affiliated Hospital of Shantou University Medical College between January 2020 and June 2022 and followed up for 6 months. All participants were assessed for ASD by 2 clinicians according to the Structured Clinical Interview for the Diagnostic and Statistical Manual of Mental Disorders, Fifth Edition (DSM-V).

### 2.2 Inclusion and Exclusion Criteria

Inclusion criteria were as follows: (1) children aged between 1 and 6 years old; (2) parents of children voluntarily participated in VB-MAPP-based intensive behavioral intervention at home for 6 months; (3) the children’s Infant-Junior Middle School Student’s Social Life Ability Scale (IJMSSSLAS) score was <10 points, or the Gesell Development Scale (Gesell) score was <75 points, or the Autism Behavior Checklist (ABC) score was <31 points; and (4) the children completed at least 2 VB-MAPP assessments.

Exclusion criteria were as follows: (1) children diagnosed with Rett syndrome, Asperger’s syndrome, other developmental disorders, or other inherited metabolic diseases; (2) children with a history of diseases that seriously affect the development of the nervous system, such as hyperbilirubinemia, kernicterus, hypoxic-ischemic encephalopathy, and epilepsy; and (3) a family history of mental retardation, epilepsy, or genetic diseases.

A total of 30 age- and sex-matched children without ASD undergoing physical examination were also recruited from the same hospital. The mental development of the children without ASD required a total and subscale Developmental Quotient (DQ) score of ≥70 on the Griffiths Developmental Scale-Chinese version (GDS-C). Children without ASD with a diagnosed neurological condition (e.g., neurodevelopmental delay) were excluded.

The study protocol complied with the Declaration of Helsinki and was approved by the Ethics Committee of the Second Affiliated Hospital of Shantou University Medical College (No. 2020-3). Parents/guardians agreed to participate using an informed consent procedure without any cost.

### 2.3 Data Collection and Measurements

Basic information about the patients was collected through a questionnaire survey. The variables of interest included the child’s sex and age (years), place of residence, siblings, average monthly household income, mother’s and father’s occupation, mother’s and father’s education level, mother’s and father’s age (years), and birth order of children. The ABC and IJMSSSLAS were completed by the child’s parents or caregivers. The Gesell scale, GDS-C, and VB-MAPP were evaluated by a health professional.

#### 2.3.1 ABC Scale

The children’s autism symptoms were assessed by the Chinese version of the 57-item ABC [30], which has been psychometrically tested for validity and reliability [31, 32]. Each item scores between 1 and 4 points, and a total score above 31 points indicates positive autism symptoms [33].

#### 2.3.2 IJMSSSLAS Scale

The children’s social life functioning was measured by the Chinese version of the IJMSSSLAS [34]. Based on the Japanese version of the children’s Infant-Junior Middle School Student’s Social Life Ability Scale (S-M scale), the IJMSSSLAS contains 132 items with good validity and reliability [35]. Each item is assigned 1 point, and the final score is converted to a standardized score that is adjusted for age. Standardized scores less than 10 indicate deficiencies in social living ability [36, 37].

#### 2.3.3 Gesell Scale

The children’s early neurological development was examined by the Chinese version of the 97-item Gesell scale [38]. Good psychometric properties have been demonstrated previously [39]. The developmental quotient (DQ) was calculated based on developmental age and actual age. A total DQ and/or sub-DQ score <75 is considered to indicate developmental delay [40].

#### 2.3.4 GDS-C Scale

The mental development of children was identified by the GDS-C [41, 42]. The GDS-C has been evaluated extensively for its psychometric properties [41, 43]. A general and subscale DQ of the GDS-C ≥70 is considered normal [44].

#### 2.3.5 Verbal Behavior Milestones Assessment and Placement Program Indicators

The children’s language, social, and other skills were assessed by the Chinese version of VB-MAPP, 2nd edition [45, 46, 47, 48], which contains 16 domains with 5 to 15 milestones per domain. Depending on the age range of the child, different developmental milestones are evaluated (level 1: 0–18 months; level 2: 18–30 months; level 3: 30–48 months). A total of 170 measurable milestones include Mand, Tact, Listener Responding, Visual Perceptual Skills and Matching-to-Sample, Independent Play, Social Behavior and Social Play, Motor Imitation, Echoic, and Spontaneous Vocal Behavior (level 1); Listener Responding by Function, Feature and Class, Intraverbal, Classroom Routines and Group Skills, and Linguistic Structure (level 2); and Reading, Writing, and Mathematics (level 3). Each milestone scores “0”, “0.5”, or “1” point, depending on performance. The maximum cumulative score is 170, with higher scores indicating better performance. The VB-MAPP has good psychometric properties [49, 50] and is widely used in different countries.

### 2.4 Intervention

All children without ASD had never received any interventions. The children with ASD received intensive home-based behavioral intervention for 6 months. According to the VB-MAPP initial assessment and the child’s family situation, an individualized intervention strategy was developed with the children’s parents. The main intervention procedure is organized into 2 parts: the physician-assisted parental intervention phase and the parent-led intervention phase.

#### 2.4.1 Physician-assisted parental intervention phase

Physicians trained the parents on how to perform ABA interventions at home, and the parents observed the physicians teaching their children using the ABA therapy (i.e., DTT and NET teaching techniques). During the 6-month intervention period, all training was conducted weekly for the first 3 months, then decreased to biweekly for the next month, and monthly for the last 2 months. Each intervention lasted 80 minutes, including 40 minutes of 1-on-1 ABA therapy between a physician and a child, and 40 minutes of ABA therapy training for the parents.

#### 2.4.2 Parent-led intervention phase

The parents were required to conduct at least 20 hours of ABA family training and upload at least 3 videos per week to the WeChat group. The physicians watched all videos and solved any operational problems that occurred in the videos. When the parents visited the physicians for the next physician-assisted parent intervention, the physicians demonstrated ABA therapy again based on the parents’ operational techniques that were shown in the previously uploaded video and asked the parents to re-perform until the technique was fully mastered. The teaching themes and contents of parent training are shown in Table 1. Relevant assessments were collected at baseline and post-intervention (Wave 1).

**Table 1.** **Teaching themes and contents of parent training**.

No	Themes	Contents of Courses
1	ASD- and ABA-related knowledges	ASD clinical manifestations, evidence-based treatments, behavior shaping, positive and negative reinf​orcem​ent/p​unish​ment,​ etc.
2	Problem behavior intervention	Eliminating problem behaviors and reinforcing good behaviors
3	ABA practices	Establishing matching relationships with children; reinforcers of discovery and utilization; instructional control establishment; aiding and assisting retreats; token utilization; data recording; etc.
4	Hands-on training in different language domains for the VB-MAPP	Hands-on training methods such as “Mand”, “Tact”, “Listener Responds”, “Visual Perceptual Skills” and “Matching-to-Sample”, etc.
5	Natural contextual generalization instruction	Generalization of abilities, such as “Mand”, “Tact”, “Listener Responds”, etc., in natural contexts

ABA, applied behavior analysis; ASD, autism spectrum disorder; VB-MAPP, Verbal Behavior Milestones Assessment and Placement Program.

### 2.5 Statistical Analysis

Statistical analyses were performed using SPSS v. 25 (IBM SPSS, IBM Corp., Armonk, NY, USA), and significance was set at *p* < 0.05.

#### 2.5.1 Sample Size Estimation

The sample size was estimated using the “power and sample size” module in Stata 15.1. Given the findings from an earlier study (pretreatment mean: 17.5 ± 14.6; 6 months posttreatment mean: 34.1 ± 24.9) [29], statistical power of 0.9, and a significance level of 99% (2-tailed), the sample size should be at least 10. With an assumed response rate of 80%, a total of 13 children should be included.

#### 2.5.2 Missing Data

The Little’s completely at random (MCAR) test criterion (including 16 domains of VB-MAPP, child’s sex, place of domicile, siblings, average monthly household income, father’s and mother’s occupation, father’s and mother’s education level, child’s age, child’s age of onset, father’s and mother’s age, and birth order of children) was met (χ^2^ = 114.238, *p* = 0.67). Multiple imputations (N = 10) were conducted to solve the missing data.

#### 2.5.3 Descriptive Analyses

Descriptive analyses were performed to understand the characteristics of the study sample. Mean ± standard deviation (SD) or median and interquartile range (IQR) were used for continuous variables, whereas percentages and numbers were used for categorical variables. Differences in the outcomes were tested by the Mann–Whitney *U* test for continuous variables and chi-squared tests (χ^2^) for categorical variables.

#### 2.5.4 Difference Analyses

To observe the effectiveness after intensive home-based behavioral intervention among children with ASD, we used Wilcoxon rank-sum tests. Mann–Whitney *U* tests were used to compare the differences in VB-MAPP total score and 16 domain scores between children with ASD at baseline, children with ASD after intervention, and children without ASD.

#### 2.5.5 Non-Inferiority Test

Owing to the lack of minimal clinically important difference (MCID) for VB-MAPP, the Vineland-II Adaptive Behavior Scales (V-ABC) with an MCID value of 2 was considered [24, 51]. A 2-sided non-inferiority test for the primary outcome was conducted by comparing the 95% confidence intervals (CIs) of independent *t*-tests between 2 groups with the predetermined MCID.

## 3. Results

### 3.1 Subjects’ Characteristics

Children with ASD (n = 85) had a median age of 2.667 years (IQR: 2.083–3.583). Sixty-nine children with ASD were boys (81.2%), and the majority of children with ASD were from 1-child families. Thirty children without ASD were included in the study, with a median age of 2.545 years (IQR: 2.148–3.580). The mean age of ASD onset was 2.70 years (SD: 0.89) for Children with ASD. Characteristics of the individuals are summarized in Table 2. No significant differences between children with ASD and children without ASD were found in child’s sex, place of domicile, siblings, average monthly household income, father’s and mother’s occupation, father’s and mother’s education level, child’s age, father’s age, and birth order of children (all *p* > 0.05), although mother’s age were significantly different (*p* = 0.017), showing a good comparability between groups.

**Table 2.** **Comparison of baseline information between children with ASD and children without ASD**.

Variable		Children with ASD (n = 85)	Children without ASD (n = 30)	χ ^2^	*p*
		N (%)	N (%)		
Sex		​	​	0.020	0.888
	Boy	69 (81.2)	24 (80.0)	​	​
	Girl	16 (18.8)	6 (20.0)	​	​
Place of domicile		​	​	1.427	0.232
	Rural	61 (71.8)	18 (60.0)	​	​
	Urban	24 (28.2)	12 (40.0)	​	​
Siblings*		​	​	1.921	0.166
	Yes	13 (15.3)	8 (26.7)	​	​
	No	72 (84.7)	22 (73.3)	​	​
Average monthly household income		​	​	1.725	0.189
	More than 1374 USD	42 (49.4)	19 (63.3)	​	​
	1374 USD and below	43 (50.6)	11 (36.7)	​	​
Father’s occupation		​	​	0.520	0.471
	Enterprises and institutions	31 (37.3)	9 (30.0)	​	​
	Freelance and unemployment	52 (62.7)	21 (70.0)	​	​
Father’s education level		​	​	0.042	0.837
	Higher vocational colleges and above	35 (41.2)	13 (43.3)	​	​
	Secondary specialized school and below	50 (58.8)	17 (56.7)	​	​
Mother’s occupation		​	​	0.409	0.522
	Enterprises and institutions	31 (36.5)	9 (30.0)	​	​
	Freelance and unemployment	54 (63.5)	21 (70.0)	​	​
Mother’s education level		​	​	0.003	0.956
	Higher vocational colleges and above	42 (49.4)	15 (50.0)	​	​
	Secondary specialized school and below	43 (50.6)	15 (50.0)	​	​
​		Median (*p*_25_–*p*_75_)	Median (*p*_25_–*p*_75_)	*Z*	*p*
Child’s age (years)		2.67 (2.08–3.58)	2.55 (2.15–3.58)	0.833	0.405
Child’s age of onset (years)		2.58 (2.08–3.33)	-	-	-
Father’s age (years)		34.00 (31.00–36.00)	35.50 (33.00–38.00)	−1.810	0.070
Mother’s age (years)		32.00 (29.00–34.00)	33.50 (31.00–35.25)	−2.383	**0.017**
Birth order of children		1.76 (1.00–2.00)	2.00 (1.00–2.00)	0.471	0.610

Bold *p* values: *p* < 0.05. ASD, Autism Spectrum Disorder. *Do you have brothers or sisters? $1.0 USD = ¥7.2 CNY, 1374 US dollars is approximately equivalent to 10,000 Chinese yuan.

### 3.2 Comparison before and after Intervention

**Fig. 1.**
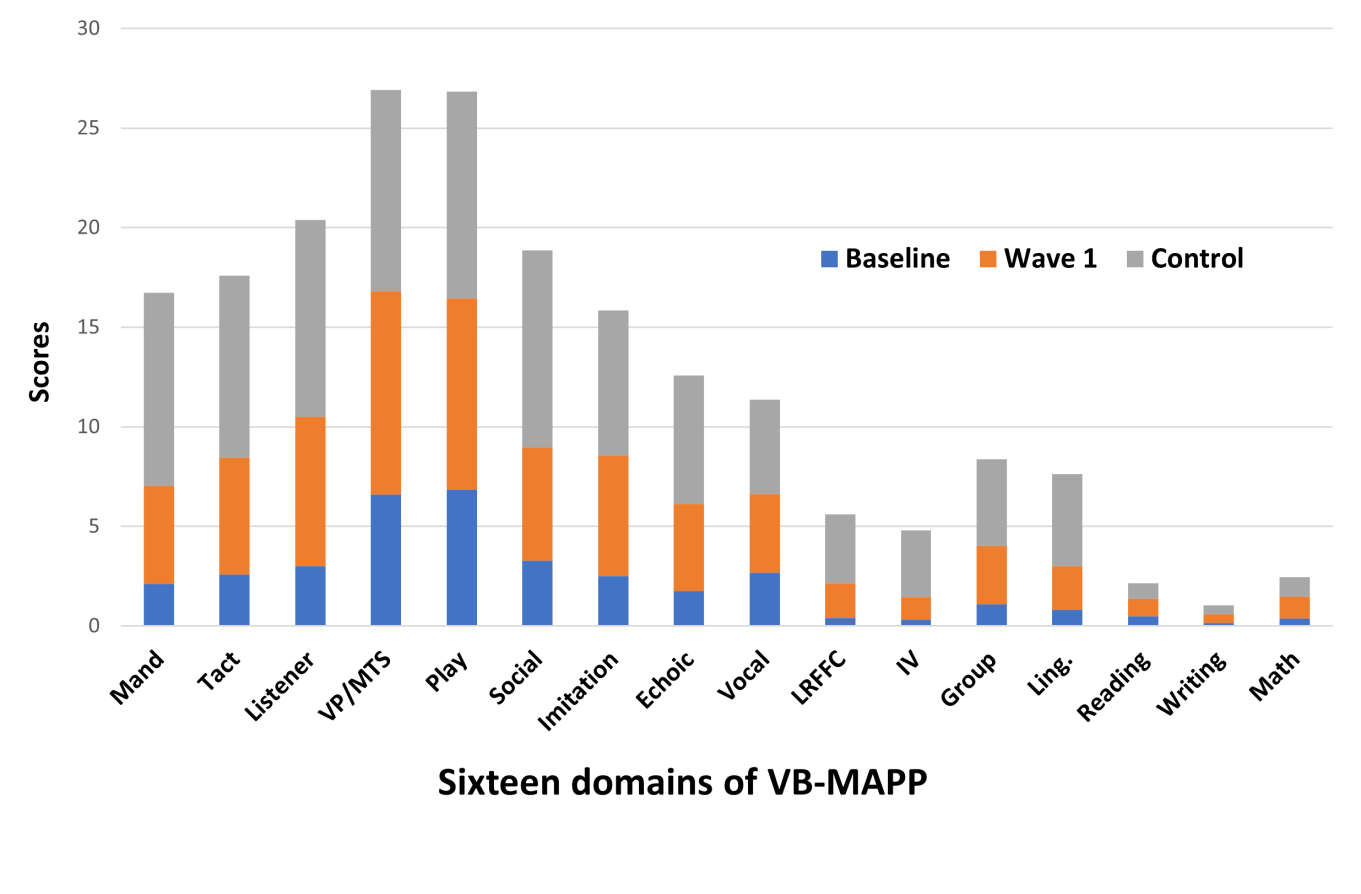
**Profile of VB-MAPP mean scores across different groups**. VB-MAPP, Verbal Behavior Milestones Assessment and Placement Program; Group, Classroom Routines and Group Skills; Imitation, Motor Imitation; IV, Intraverbal; Ling., Linguistic Structure; Listener, Listener Responding; LRFFC, Listener Responding by Function, Feature and Class; Math, Mathematics; Play, Independent Play; Social, Social Behavior and Social Play; Vocal, Spontaneous Vocal Behavior; Feature and Class; VP/MTS, Visual Perceptual Skills and Matching-to-Sample.

As shown in Table 3 and Fig. 1, a significant improving effect of intensive home-based behavioral intervention was found between children with ASD at baseline and children with ASD at Wave 1 in terms of total VB-MAPP score (*p* < 0.001), Mand score (*p* < 0.001), Tact score (*p* < 0.001), Listener score (*p* < 0.001), VP/MTS score (*p* < 0.001), Play score (*p* < 0.001), Social score (*p* < 0.001), Imitation score (*p* < 0.001), Echoic score (*p* < 0.001), Vocal score (*p* < 0.001), LRFFC score (*p* < 0.001), IV score (*p* < 0.001), Group score (*p* < 0.001), Ling. score (*p* < 0.001), Reading score (*p* < 0.001), Writing score (*p* < 0.001), and Math score (*p* < 0.001), indicating that after the intervention, the abilities of children with ASD were improved, as measured by the VB-MAPP.

**Table 3.** **Comparison of total and 16 domains of VB-MAPP scores between children with ASD at baseline and at Wave 1**.

Variables	Children with ASD Baseline (n = 85)		Children with ASD Wave 1 (n = 85)		*Z*	*p*
	Mean	SD	Mean	SD		
Mand	2.129	3.11	4.924	3.74	−6.357	< **0.001**
Tact	2.600	3.42	5.847	3.89	−6.636	< **0.001**
Listener	2.994	3.79	7.512	3.95	−7.085	< **0.001**
VP/MTS	6.606	3.31	10.218	2.74	−7.526	< **0.001**
Play	6.859	2.61	9.588	2.53	−7.187	< **0.001**
Social	3.288	2.06	5.694	2.61	−7.211	< **0.001**
Imitation	2.506	3.18	6.047	2.96	−7.067	< **0.001**
Echoic	1.765	2.84	4.371	3.25	−6.475	< **0.001**
Vocal	2.659	1.64	3.982	1.20	−6.113	< **0.001**
LRFFC	0.412	1.09	1.735	2.11	−5.930	< **0.001**
IV	0.312	0.82	1.147	1.66	−4.909	< **0.001**
Group	1.106	2.01	2.929	3.11	−5.599	< **0.001**
Ling.	0.818	1.48	2.182	2.03	−6.214	< **0.001**
Reading	0.471	1.22	0.906	1.58	−3.641	< **0.001**
Writing	0.153	0.60	0.429	1.01	−3.746	< **0.001**
Math	0.365	0.90	1.106	1.49	−4.977	< **0.001**
Total score	33.624	29.52	70.435	40.36	−8.008	< **0.001**

SD, Standard Deviation; ASD, Autism Spectrum Disorder; VB-MAPP, Verbal Behavior Milestones Assessment and Placement Program; Group, Classroom Routines and Group Skills; Imitation, Motor Imitation; IV, Intraverbal; Ling., Linguistic Structure; Listener, Listener Responding; LRFFC, Listener Responding by Function, Feature and Class; Math, Mathematics; Play, Independent Play; Social, Social Behavior and Social Play; Vocal, Spontaneous Vocal Behavior; VP/MTS, Visual Perceptual Skills and Match​ing-t​o-Sam​ple. B​old *p* values: *p* < 0.05.

### 3.3 Intervention Evaluation

Compared with children without ASD, children with ASD at baseline had lower VB-MAPP total scores and 15-dimensional scores (all, *p* < 0.05), except for math ability (*p* = 0.149), suggesting that many of the abilities of children with ASD were inferior to those of healthy children. After intensive home-based behavioral intervention, there was no significant difference between children with ASD at Wave 1 and children without ASD in the VP/MTS score (*p* = 0.699), Play score (*p* = 0.189), Group score (*p* = 0.100), Reading score (*p* = 0.221), Writing score (*p* = 0.557), and Math ability score (*p* = 0.244), while total scores (*p* = 0.002), Mand score (*p* < 0.001), Tact score (*p* < 0.001), Listener score (*p* = 0.005), Social score (*p* < 0.001), Imitation score (*p* = 0.032), Echoic score (*p* = 0.002), Vocal score (*p* < 0.001), LRFFC score (*p* = 0.001), IV score (*p* < 0.001), and Ling. score (*p* < 0.001) among children with ASD at Wave 1 remained lower than those of children without ASD (see Table 4 and Fig. 1).

**Table 4.** **Comparison of total and 16 domains of VB-MAPP scores between children with ASD and children without ASD**.

Variables	Children with ASD Baseline (n = 85)		Children without ASD (n = 30)		Children with ASD Wave 1 (n = 85)		Z*	p*	*Z* ^#^	*p* ^#^
	Mean	SD	Mean	SD	Mean	SD				
Mand	2.129	3.11	9.683	3.08	4.924	3.74	−7.264	< **0.001**	−5.544	< **0.001**
Tact	2.600	3.42	9.150	2.99	5.847	3.89	−6.711	< **0.001**	−4.000	< **0.001**
Listener	2.994	3.79	9.883	3.11	7.512	3.95	−6.666	< **0.001**	−2.803	**0.005**
VP/MTS	6.606	3.31	10.083	3.28	10.218	2.74	−4.476	< **0.001**	−0.386	0.699
Play	6.859	2.61	10.383	2.95	9.588	2.53	−5.063	< **0.001**	−1.315	0.189
Social	3.288	2.06	9.883	3.20	5.694	2.61	−7.483	< **0.001**	−5.569	< **0.001**
Imitation	2.506	3.18	7.300	2.35	6.047	2.96	−6.184	< **0.001**	−2.144	**0.032**
Echoic	1.765	2.84	6.450	2.66	4.371	3.25	−6.600	< **0.001**	−3.140	**0.002**
Vocal	2.659	1.64	4.733	0.77	3.982	1.20	−5.732	< **0.001**	−3.740	< **0.001**
LRFFC	0.412	1.09	3.467	2.63	1.735	2.11	−7.099	< **0.001**	−3.407	**0.001**
IV	0.312	0.82	3.333	2.65	1.147	1.66	−7.088	< **0.001**	−4.336	**0.001**
Group	1.106	2.01	4.333	4.43	2.929	3.11	−3.705	< **0.001**	−1.647	0.100
Ling.	0.818	1.48	4.617	2.52	2.182	2.03	−7.033	< **0.001**	−4.413	< **0.001**
Reading	0.471	1.22	0.767	1.06	0.906	1.58	−3.468	< **0.001**	−1.225	0.221
Writing	0.153	0.60	0.467	0.81	0.429	1.01	−2.509	**0.012**	−0.587	0.557
Math	0.365	0.90	0.967	1.86	1.106	1.49	−1.444	0.149	−1.166	0.244
Total score	33.624	29.52	95.500	36.47	70.435	40.36	−6.540	< **0.001**	−3.077	**0.002**

Bold *p* values: *p* < 0.05; SD, Standard Deviation; ASD, Autism Spectrum Disorder; VB-MAPP, Verbal Behavior Milestones Assessment and Placement Program; Group, Classroom Routines and Group Skills; Imitation, Motor Imitation; IV, Intraverbal; Listener, Listener Responding; Ling., Linguistic Structure; LRFFC, Listener Responding by Function, Feature and Class; Math, Mathematics; Play, Independent Play; Social, Social Behavior and Social Play; Vocal, Spontaneous Vocal Behavior; VP/MTS, Visual Perceptual Skills and Matching-to-Sample. *represents baseline children with ASD versus children without ASD. ^#^represents children with ASD after intervention (Wave 1) versus children without ASD.

### 3.4 Non-Inferiority Test

With the noninferiority margin equal to 2, the observed difference between children with ASD and those without ASD supported noninferiority in the VP/MTS score (*p* = 0.001), Play score (*p* = 0.034), Reading score (*p* < 0.001), and Writing score (*p* < 0.001), but not Group score (*p* > 0.05), indicating that after the intervention, children with ASD had reached similar levels of competence in these dimensions compared with children without ASD.

## 4. Discussion

To our best knowledge, this is the first report to compare the effectiveness of VB-MAPP-based family-intensive behavioral intervention between children with ASD and those without ASD. This study had several main findings. (1) Children without ASD had significantly higher VB-MAPP total scores and 15-dimensional scores than children with ASD at baseline, except for the math ability score. (2) After 6 months of VB-MAPP-based family-intensive behavioral intervention, VB-MAPP total score and 16-dimensional scores showed significant improvement in children with ASD. (3) Post-intervention, the effectiveness of VB-MAPP-based family-intensive behavioral intervention for children with ASD was non-inferior to that for children without ASD in some VB-MAPP dimension scores (i.e., VP/MTS, play, reading, and writing).

Previous studies have demonstrated that ABA principles and language behavior methods in children with ASD can effectively enhance their language skills, as measured by the VB-MAPP assessment tool [52, 53], which is in agreement with our study. The key principle of ABA is to use the psychological principles of learning theory to change common behaviors in children with ASD [54]. Owing to the large number of highly plastic neuronal connections present in the early stages of life [55], early intensive behavior intervention can minimize intervention costs and maximize the benefits for children with ASD [27]. However, one study found that children with ASD in the motor and vocal imitation assessment (MVIA) treatment group outperformed the VB-MAPP comparison group [56], but a major intervention strategy in this study was imitation intervention based on DTT.

Parents can continue to provide intervention over a long period of time due to their long-term presence with their children [57]. There is accumulating evidence regarding the effectiveness of various family training programs for children with ASD. One study indicated that parental training for children with ASD had a mild to moderate effect on ASD symptoms [58]. Another study also revealed that parent training can be useful not only for children with ASD, but for children without ASD who had daily dysfunction [59]. Importantly, a randomized controlled trial (RCT) study found that parent-implemented interventions are effective in enhancing the social communication ability and family quality of life for children with ASD [60]. These findings from family interventions were similar to those of our family intervention-focused studies. Our study also extended the family intervention program further by adding family theory training combined with ABA therapy and revealed a positive effect. Wang Ting *et al*. in 2021 [61] showed that after 1 year of intervention, the VB-MAPP score in children with ASD undergoing family theory and practical training combined with ABA therapy was higher than that of the family theory training only group. The latter scored higher than the purely institutional ABA therapy group, indicating that theoretical and practical training for parents combined with ABA therapy is an efficient and feasible intervention strategy.

The findings in our study demonstrated that the VB-MAPP-based family-intensive behavioral intervention has a substantial effect on visual perception ability, independent gaming ability, and language development ability. Visual perception ability can reflect a child’s cognitive function and is one of the main components of cognitive tests [62]. One study also revealed that pivotal response treatment parent training could improve the language and cognitive function [63]. One major reason is that children with ASD are more willing to cooperate with training for visual perception. Therefore, the parents might conduct more visual matching training. A RCT study identified that parent-involved intervention can effectively improve the independent play ability of children with ASD [64]. The game ability in VB-MAPP requires children to learn, imitate, and create on the basis of paying attention to the people around them, and gradually acquire the ability to play independently. The intervention we studied can encourage parents and children to have more effective interaction time and teaching opportunities and also promote the improvement of children’s independent play ability during game interaction with other children. Reading, mathematics, and writing are classified as academic abilities in the VB-MAPP assessment. Multiple studies also found that parental involved interventions could impact academic abilities in children with ASD [65, 66, 67]. A possible reason is that a large proportion of children with ASD have normal or high intelligence [68].

The strength of this study is that we included a group of controls without ASD, who were measured using the VB-MAPP, and we used a low-cost and effective intervention. However, some limitations need to be acknowledged. First, the sample size was limited; future studies should include a larger sample size. Second, it is unclear whether children with ASD received additional interventions, thus the interpretation of the results should be cautious. Third, owing to the lack of post-intervention assessments of symptomatology or developmental indicators for children with ASD, relevant results on these indicators are inconclusive. Fourth, the long-term effects of VB-MAPP-based family-intensive behavioral intervention were not evaluated, and further exploration is warranted.

## 5. Conclusions

The findings provide evidence that children with ASD’s language, social, and multifaceted skills can be strengthened using VB-MAPP-based family-intensive behavioral intervention. The Verbal Behavior Milestones Assessment and Placement Program-based family intensive behavioral intervention has potential for large-scale implementation, although further evidence from large, multi-center, randomized controlled trials is required.

## Availability of Data and Materials

The data that support the findings of this study are available on request from the first/corresponding author.
